# Impact of comorbid conditions on participation in an organised colorectal cancer screening programme: a cross-sectional study

**DOI:** 10.1186/s12885-017-3516-x

**Published:** 2017-08-07

**Authors:** Carolina Guiriguet, Guillem Pera, Antoni Castells, Pere Toran, Jaume Grau, Irene Rivero, Andrea Buron, Francesc Macià, Carmen Vela-Vallespín, Mercedes Vilarrubí-Estrella, Mercedes Marzo-Castillejo

**Affiliations:** 1 Family Medicine Department, Catalan Institute of Health, Santa Coloma de Gramenet, Spain; 2 Unitat de Suport a la Recerca Metropolitana Nord, Institut Universitari d’Investigació en Atenció Primària Jordi Gol (IDIAP Jordi Gol), Mataró, Spain; 3 Gastroenterology Department, Hospital Clinic, University of Barcelona, August Pi i Sunyer Biomedical Research Institute (IDIBAPS), Biomedical Research Networking Center Consortium in Hepatic and Digestive diseases (CIBEREHD), Barcelona, Spain; 4 Preventive Medicine and Hospital Epidemiology Department, Hospital Clinic, University of Barcelona, Barcelona, Spain; 5 Family Medicine Department, Catalan Institute of Health, Barcelona, Spain; 6 Preventive Medicine and Epidemiology Department, Hospital del Mar Medical Research Institute, Barcelona, Spain; 7 Unitat de Suport a la Recerca Metropolitana Sud, Institut Universitari d’Investigació en Atenció Primària Jordi Gol (IDIAP Jordi Gol), Cornellà de Llobregat, Spain; 8 Catalan Institute of Health, Gotic Primary Care Center, Passatge de la Pau, 1, 08002 Barcelona, Spain

**Keywords:** Colorectal neoplasm, Early detection of cancer, Mass screening, Primary health care, Chronic disease, Comorbidity, Public health

## Abstract

**Background:**

There is controversy regarding how comorbidity impacts on colorectal cancer screening, especially in the context of organised programmes. The aim of this study is to assess the effect of comorbidities on participation in the Barcelona population-based colorectal cancer screening programme (BCCSP).

**Methods:**

Cross-sectional study carried out in ten primary care centres involved in the BCCSP. Individuals aged 50 to 69, at average risk of colorectal cancer, who were invited to participate in the first round of the faecal immunochemical test-based BCCSP were included (2011–2012). The main variable was participation in the BCCSP. Comorbidity was assessed by clinical risk group status. Other adjusting variables were age, sex, socioeconomic deprivation, visits to primary care, smoking, alcohol consumption and body mass index. Logistic regression models were used to test the association between participation in the programme and potential explanatory variables. The results were given as incidence rate ratios (IRR) and their 95% confidence intervals (CI).

**Results:**

Of the 36,208 individuals included, 17,404 (48%) participated in the BCCSP. Participation was statistically significantly higher in women, individuals aged 60 to 64, patients with intermediate socioeconomic deprivation, and patients with more medical visits. There was a higher rate of current smoking, high-risk alcohol intake, obesity and individuals in the highest comorbidity categories in the non-participation group. In the adjusted analysis, only individuals with multiple minor chronic diseases were more likely to participate in the BCCSP (IRR 1.14; 95% CI [1.06 to 1.22]; *p* < 0.001). In contrast, having three or more dominant chronic diseases was associated with lower participation in the screening programme (IRR 0.76; 95% CI [0.65 to 0.89]; *p* = 0.001).

**Conclusions:**

Having three or more dominant chronic diseases, was associated with lower participation in a faecal immunochemical test-based colorectal cancer screening programme, whereas individuals with multiple minor chronic diseases were more likely to participate. Further research is needed to explore comorbidity as a cause of non-participation in colorectal cancer screening programmes and which individuals could benefit most from colorectal cancer screening.

## Background

In Western countries, colorectal cancer has the highest incidence rate of all cancers and is the second leading cause of cancer death in both sexes [1]. Evidence from several studies has demonstrated that colorectal cancer screening is effective and cost-effective in terms of reducing disease-specific mortality in average-risk populations through the detection of early-stage adenocarcinomas and the detection and removal of adenomatous polyps [2]. Recommended colorectal cancer screening strategies fall into two categories: stool tests that primarily detect cancer, which involve the detection of occult blood or exfoliated DNA; and structural exams, which are effective in detecting both cancer and premalignant lesions and include flexible sigmoidoscopy, colonoscopy, and computed tomography colonography [3]. Of these techniques, the guaiac faecal occult blood test and, more recently, the faecal immunochemical test (FIT) are the ones that are used most frequently in European colorectal cancer screening programmes [4]. In contrast, colonoscopy is the dominant screening modality in North America [3]. Following European Union recommendations [5], colorectal cancer screening programmes have been implemented progressively in Spain in recent years and have involved men and women aged 50 to 69 at average risk of developing colorectal cancer. Nonetheless, participation in these programmes, with the exception of some regions, has not reached the desired rate [6]. Several factors influence participation in cancer screening. The most significant factors are factors related to the healthcare system and intrapersonal factors [7]. The former can be minimised as an obstacle in publicly organised programmes [4]. The latter include social, cultural and psychological issues (e.g. knowledge about a specific disease, the benefits of screening, and the perceived risk, benefits, barriers) which may, in turn, interact with one other in a complex way [8].

Older age is associated with an increase in the prevalence of cancer and other chronic conditions or comorbidities, and questions remain about the interactions between comorbidity and cancer screening participation [9]. A recent systematic review and meta-analysis, focusing on breast and cervical cancer, reported that results from high quality studies suggested that women with a comorbidity are less likely to participate in breast - and possibly cervical - cancer screening, although a definitive conclusion could not be drawn [10]. However, few studies have focused on comorbid conditions and participation on colorectal cancer screening [11–14]. On the other hand, comorbidities influence the cost effectiveness of screening, which depends on a patient’s current diseases, individual background risk of developing colorectal cancer, the previous screening history frequency and life expectancy [15, 16]. The higher risk of death from competing diseases at advanced ages and the risk of screening induced harms (i.e. colonoscopy-related complications and over-diagnosis and over-treatment of colorectal cancer), which increases with older age, tend to cancel out the benefits of screening. The results of a study on the appropriate age to stop colonoscopy screening (i.e., the maximum age at which screening is cost-effective) given sex, race, screening history, background risk of colorectal cancer and comorbidity status showed that, while having fewer comorbidities was associated with cost-effective screening at older ages, sex and race had only a small effect on the appropriate age to stop screening [15]. Authors concluded that colorectal cancer screening could be more effective and cost effective if each patient’s individual factors were taken into account [15].

In this scenario, learning about how existing comorbidities affect key performance indicators for colorectal cancer screening programmes must be a priority. In the Barcelona Colorectal Cancer Screening Programme (BCCSP), men and women aged 50 to 69 are invited to a FIT every 2 years [17]. Individuals receive a mailed invitation letter along with an explanatory leaflet. The FIT is distributed through the community pharmacies involved in the programme, and participants with a positive test are invited to undergo a colonoscopy. Depending on the result of colonoscopy, patients are referred to primary care or specialists for follow-ups, or invited to re-enter the programme. Both primary care professionals and pharmacists receive a specific training session at the beginning of each screening round, during which they learn about the implementation of the programme. The role of primary care professionals is to help the programme to narrow down the target population, identifying individuals with the exclusion criteria, and to promote participation. In the context of the BCCSP, this study is part of the Colo-alert [18], a cluster randomised clinical trial in primary care. The aim of this ad hoc analysis is to assess the association between comorbidities and colorectal cancer screening uptake in the context of an organised FIT-based programme.

## Methods

### Study setting and population

**Fig. 1:**
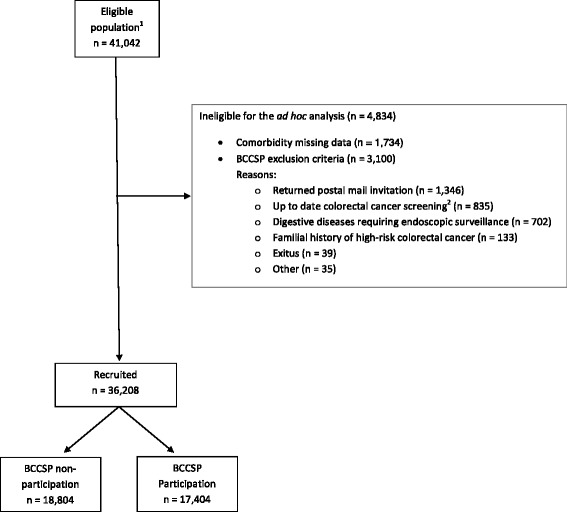
Flowchart of the study. ^1^Corresponding to individuals involved in the cluster randomized controlled trial Colo-alert (see reference [19]). ^2^Colonoscopy performed in the last five years or faecal occult blood test in the last two years. BCCSP: Colorectal Cancer Screening Programme of Barcelona

Cross-sectional study carried out at 10 Barcelona primary care centres involved in the BCCSP from July 2011 to May 2012. The inclusion criterion for this ad hoc analysis was individuals eligible to participate in the first round of the BCCSP (i.e. men and women aged 50 to 69 at average risk of colorectal cancer) included in the Colo-alert trial (*n* = 41,042) [18]. The exclusion criterion for this ad hoc analysis was the presence of BCCSP exclusion criteria (*n* = 3100). Individuals with missing data on comorbidities were excluded (*n* = 1734; 4.6%). In the end, a total of 36,208 individuals were included in the study (Fig. 1).

The Colo-alert trial was a cluster randomised clinical trial (RCT) in primary care, that evaluated the effectiveness of an alert in the electronic medical record (intervention group) to encourage colorectal cancer screening participation compared to usual care (control group) in which primary care professionals were involved in order to improve colorectal cancer screening participation [19]. The BCCSP was launched in December 2009 and the first round lasted until May 2012. The target population comprised 197,795 individuals invited based on the primary care centre they had been assigned to. More specifically, the specific cohort of individuals enrolled in the cluster-RCT included those individuals assigned to the last 10 primary care centres that were invited from July 2011 to May 2012. These centres, run by the Catalan Institute of Health, were waiting to start the first round of the BCCSP at the time the intervention was started. Of the 11 primary care centres invited to take part in the RCT (*n* = 148 general practitioners -GP- and 57,020 patients), one refused to participate (*n* = 18 GP and 5953 patients). Subsequently, 10,025 patients who did not have a GP assigned to a participating centre at the start of the study were excluded. Therefore, they were not eligible to receive the RCT intervention. Following the above exclusions, we considered the appropriateness of including the entire population - 130 GP with 41,042 individuals.

### Variables and data source

The main dependent variable was participation in the BCCSP within 1 year of the date of invitation.

The main explanatory variable was comorbidity according to clinical risk group (CRG) status [20]. The Catalan Institute of Health incorporated the CRG tool as a model for grouping morbidities. Its use involves a computerised calculation based on a patient’s basic information as collected in their electronical medical record (age, sex, International Classification of Diseases 10) and the assignment and visualisation of the CRG stratification results for each user in the computerised medical record used by GP. The CRG status includes the following nine categories:

#### Healthy (CRG 1)

Absence of any primary chronic diseases or significant acute disease episodes or episode procedure categories.

#### History of significant acute disease (CRG 2)

Presence within the last 6 months of one or more significant acute disease episode categories (i.e., chest pain) or significant episode procedure categories with no primary chronic diseases present.

#### Single minor chronic disease (CRG 3)

Presence of a single minor primary chronic disease (i.e., migraine headache, hearing loss, hyperlipidaemia).

#### Minor chronic disease in multiple organ systems (CRG 4)

Presence of two or more minor primary chronic diseases.

#### Single dominant or moderate chronic disease (CRG 5)

presence of a single dominant (i.e., congestive heart failure, diabetes) or moderate (i.e., asthma, epilepsy) primary chronic disease.

#### Significant chronic disease in multiple organ systems (CRG 6)

Presence of two or more primary chronic diseases, of which at least one is a dominant or moderate primary chronic disease.

#### Dominant chronic disease in three or more organ systems (CRG 7)

Presence of three or more dominant primary chronic diseases.

#### Dominant and metastatic malignancies (CRG 8)

A malignancy that dominates the medical care required (i.e. brain malignancy) or a metastatic non-dominant malignancy (i.e., prostate malignancy).

#### Catastrophic conditions (CRG 9)

Long-term dependency on a medical technology (i.e., dialysis, respirator) and life-defining chronic diseases or conditions that dominate the medical care required (i.e., persistent vegetative state, cystic fibrosis, acquired immunodeficiency syndrome, history of heart transplant).

Other adjusting explanatory variables were age, sex, body mass index (BMI), smoking status (never smoked, ex-smoker, current smoker), alcohol consumption (non-drinker, low-risk drinker, high-risk drinker), socioeconomic deprivation index, number of visits to primary care during the follow-up period (1 year) and the study group to which individuals were allocated in the Colo-alert study [19] (control or intervention). BMI was grouped into three categories comprising low-normal weight (<25 kg/m^2^), overweight (25–29.9 kg/m^2^) and obesity (≥30 kg/m^2^). A high-risk alcohol drinker was someone who drinks regularly (i.e. more than 4 standard drinks per day for men, or more than 2 standard drinks for women) or who binge drinks (defined as drinking at least 5 standard drinks for men or 4 for women, in one sitting). The socioeconomic deprivation index was based on the results of the Medea Project [21], using five simple ecological indicators from the 2001 census (unemployment, manual workers, casual workers, poor level of education and poor level of education among young people) [21]. For the city of Barcelona, five socioeconomic level groups were constructed based on census tract quintiles for the city, with the first quintile category (Q1) representing the least deprived, to the fifth quintile category (Q5) representing the most deprived.

The information on the target population for the colorectal cancer screening programme was taken from the Central Registry of Insured People in the Primary Healthcare Information System. At the beginning of the RCT the BCCSP technical office provided a list of individuals who had been invited to participate in the programme and their corresponding primary care centre. All individuals’ characteristics were obtained from the patients’ electronic medical record and provided by the Catalan Institute of Health’s Primary Care Services Information System at the beginning of the study; information regarding an individual’s participation in the screening programme was provided by the BCCSP. Data anonymity was guaranteed throughout the whole study process.

### Statistical analysis

All the individuals eligible for the study were included, so no sampling was performed. Results are expressed as a frequency and percentage for categorical variables, and mean and standard deviation for continuous variables. Statistically significant differences (*p* < 0.05) between participants and non-participants in the program were evaluated using chi-squared tests (categorical variables) and Student’s t-test (continuous variables). Bivariate and multivariate Poisson regression was used to test the association between participation in the program and potential explanatory variables, individually and mutually adjusted, and the results were expressed in incidence rate ratios (IRR) and their 95% confidence intervals (CI). Exposure was set constant for all the participants. No imputation of missing values was performed. Most of the relevant adjusting variables had no missing values. The CRG variable was analysed using the original nine categories and also grouping them into four categories (i.e., healthy or acute disease, minor chronic disease, dominant chronic disease, malignancies or catastrophic diseases). All analyses were bilateral and performed with Stata v14 software.

## Results

Of the initial population, 3100 subjects were excluded as they met the exclusion criteria for participation in the BCCSP and 1734 were excluded as there was no comorbidity information available for them. In comparison with the group of patients included in the study, in the group of patients with no information on the variable CRG status we observed a greater proportion of men (54% vs. 46%), of individuals in the fifth deprivation quintile (25% vs. 19%), of individuals who did not attend their health centre in the last year (55% vs. 23%) and of smokers (34% vs. 24%). The differences were statistically significant for all the variables mentioned (*p* < 0.001). In the end, a total of 36,208 individuals were included in the study (Fig. 1); the average age was 59 years and 54% were women. Healthy individuals constituted 22.1% of the sample, and just over half of individuals had one (26.8%) or two (29.4%) dominant chronic diseases. 46.2% of the study population had used primary healthcare services five times or more within a 1 year period, and almost a quarter of individuals had not used them once (22.8%). The rest of individuals’ baseline characteristics are provided in Table 1.

**Table 1:** Participation in the colorectal cancer screening programme by individuals’ characteristics

	Total		Colorectal cancer screening programme				*p*
			Non-participation		Participation		
	n	%	n	%	n	%	
Sex							<0.001
Male	16,650	46.0%	9059	54.4%	7591	45.6%	
Female	19,558	54.0%	9745	49.8%	9813	50.2%	
Age, years (mean, SD)	58.8	5.5	58.5	5.6	59.1	5.5	<0.001
Age groups (years)							<0.001
50–54	10,561	29.2%	5898	55.8%	4663	44.2%	
55–59	9492	26.2%	4961	52.3%	4531	47.7%	
60–64	8757	24.2%	4215	48.1%	4542	51.9%	
65–69	7398	20.4%	3730	50.4%	3668	49.6%	
Socioeconomic deprivation index (quintiles)							<0.001
Q1	6665	18.4%	3732	56.0%	2933	44.0%	
Q2	6949	19.2%	3551	51.1%	3398	48.9%	
Q3	6969	19.2%	3293	47.3%	3676	52.7%	
Q4	6963	19.2%	3482	50.0%	3481	50.0%	
Q5	6397	17.7%	3667	57.3%	2730	42.7%	
Unknown	2265	6.3%					
Visits to primary care							<0.001
None	8248	22.8%	5406	65.5%	2842	34.5%	
1–2	5880	16.2%	3296	56.1%	2584	43.9%	
3–4	5369	14.8%	2604	48.5%	2765	51.5%	
> =5	16,711	46.2%	7498	44.9%	9213	55.1%	
CRG status							<0.001
Healthy	8020	22.1%	4858	60.6%	3162	39.4%	
Acute disease	64	0.2%	38	59.4%	26	40.6%	
Minor chronic disease	4090	11.3%	2080	50.9%	2010	49.1%	
Multiple minor chronic diseases	2622	7.2%	1083	41.3%	1539	58.7%	
Single dominant chronic disease	9695	26.8%	4922	50.8%	4773	49.2%	
Two dominant chronic disease	10,629	29.4%	5205	49.0%	5424	51.0%	
Three or more dominant chronic disease	452	1.2%	270	59.7%	182	40.3%	
Malignancies	406	1.1%	219	53.9%	187	46.1%	
Catastrophic condition	230	0.6%	129	56.1%	101	43.9%	
Collapsed CRG							<0.001
Healthy or acute disease	8084	22.3%	4896	60.6%	3188	39.4%	
Minor chronic diseases	6712	18.6%	3163	47.1%	3549	52.9%	
Dominant chronic diseases	20,776	57.4%	10,397	50.0%	10,379	50.0%	
Malignancies or catastrophic condition	636	1.7%	348	54.7%	288	45.3%	
Smoking status							<0.001
Never smoker	12,464	34.4%	5735	46.0%	6729	54.0%	
Former smoker	7354	20.3%	3260	44.3%	4094	55.7%	
Current smoker	6121	16.9%	3536	57.8%	2585	42.2%	
Unknown	10,269	28.4%					
Alcohol consumption							<0.001
Non-drinker	14,661	40.5%	7103	48.4%	7558	51.6%	
Low-risk drinker	8402	23.2%	3866	46.0%	4536	54.0%	
High-risk drinker	656	1.8%	365	55.6%	291	44.4%	
Unknown	12,489	34.5%					
Body mass index (kg/m2)							<0.001
< 25	5187	14.3%	2466	47.5%	2721	52.5%	
25–29.9	8217	22.7%	3737	45.5%	4480	54.5%	
≧30	6330	17.5%	3068	48.5%	3262	51.5%	
Unknown	16,474	45.5%					
Study group^a^							<0.001
Control	17,072	47.1%	9041	53.0%	8031	47%	
Intervention	19,136	52.9%	9763	51.0%	9373	49%	
Total	36,208	100%	18,804	52%	17,404	48%	

*SD* standard deviation, *CRG* Clinical Risk Group

^a^Study group of the Colo-alert cluster randomised controlled trial

Overall, 17,404 (48%) individuals participated in the BCCSP. The bivariate analysis showed a statistically significant higher uptake in females, subjects aged 60 to 64, individuals in the third socioeconomic deprivation quintile, and those who attended their primary care centre three or more times, especially in case of five or more visits (Table 1). There was a higher proportion of current smoking, high-risk alcohol intake or obesity in the non-participation group compared to the participation group (Table 1). Participation in the BCCSP was higher in individuals with existing multiple minor chronic diseases (58.7%) compared to those in the other CRG categories (*p* < 0.001) (Table 1). On the other hand, there was a lower proportion of participants classified as healthy (39.4%) and in the acute disease category (40.6%) in the participation group compared to those who did not participate in the screening. This lower participation was also found in individuals in the categories made up of the individual’s with the most comorbidities, especially in the case of three or more dominant chronic diseases (40.3%), malignancies (46.1%) or catastrophic conditions (43.9%) (*p* < 0.001) (Table 1).

In the multivariate logistic regression analysis, this participation trend observed in bivariate analysis was maintained (Table 2). Nevertheless, after adjusting for sex, age, socioeconomic deprivation index, number of visits and study group, only individuals with multiple minor chronic diseases were more likely to participate in the BCCSP (IRR 1.14; 95% CI [1.06 to 1.22]; *p* < 0.001) (Table 2). In contrast, having more than one dominant chronic disease was associated with lower adherence to the screening programme, although this only reached statistical significance with three or more chronic diseases (IRR 0.76; 95% CI [0.65 to 0.89]; *p* = 0.001) (Table 2). Adjusting for the socioeconomic deprivation index reduced the analysis sample from 36,208 to 33,943. However, univariate results for CRG status using the reduced sample were virtually the same (data not shown). Additional adjustment for smoking, alcohol and body mass index reduced the sample to 16,294 individuals due to missing values for these three variables. In any case, the IRR were very similar to those obtained with the first set of adjusting variables, but with wider 95% confidence intervals (data not shown).

**Table 2:** Multivariate multinomial regression analysis for participation in the colorectal cancer screening programme by clinical risk groups

	Unadjusted (*n* = 36,208)				Adjusted^b^ (*n* = 33,943)			
	IRR	IC95%		p	IRR	IC95%		p
CRG status								
Healthy^a^	1	-	-	-	1	-	-	-
Acute disease	1.03	0.70	1.52	0.879	0.73	0.49	1.11	0.142
Minor chronic disease	**1.25**	**1.18**	**1.32**	**<0.001**	1.05	0.99	1.12	0.119
Multiple minor chronic diseases	**1.49**	**1.40**	**1.58**	**<0.001**	**1.14**	**1.06**	**1.22**	**<0.001**
Single dominant chronic idisease	**1.25**	**1.19**	**1.31**	**<0.001**	0.98	0.93	1.04	0.509
Two dominant chronic disease	**1.29**	**1.24**	**1.35**	**<0.001**	0.95	0.90	1.01	0.087
Three or more dominant chronic disease	1.02	0.88	1.19	0.782	**0.76**	**0.65**	**0.89**	**0.001**
Malignancies	**1.17**	**1.01**	**1.35**	**0.039**	0.87	0.74	1.01	0.067
Catastrophic condition	1.11	0.91	1.36	0.286	0.90	0.74	1.10	0.310
Collapsed CRG								
Healthy or acute disease^a^	1	-	-	-	1	-	-	-
Minor chronic diseases	**1.34**	**1.28**	**1.41**	**<0.001**	**1.09**	**1.03**	**1.15**	**0.002**
Dominant chronic diseases	**1.27**	**1.22**	**1.32**	**<0.001**	0.97	0.92	1.02	0.186
Malignancies or catastrophic condition	**1.15**	**1.02**	**1.30**	**0.025**	0.88	0.78	1.00	0.055

Statistically significant differences are showed in bold (p < 0.05).

*FIT* Faecal immunochemical test, *IRR* Incidence Rate Ratio, *CRG* Clinical Risk Group

^a^Reference category

^b^Adjusted by sex, age, socioeconomic deprivation index, number of visits, study group of the Colo-alert cluster randomised controlled trial

## Discussion

### Summary

This study assessed the impact of comorbidity on FIT-based colorectal cancer screening programme uptake in men and women aged 50 to 69. Individuals with multiple minor chronic diseases were more likely to participate in the screening programme compared to healthy subjects. In contrast, subjects who had three or more dominant chronic diseases were less likely to participate in the programme.

### Comparison with existing literature

The total crude participation rate in the BCCSP (48%) observed in this study reached the standard of more than 45% referred to in the European guidelines [4]. It was higher than the average colorectal cancer screening rates in Spain (43.8%) [6], but lower than the results for the programme in the Basque Country (64.3%) [22] in Spain and other European colorectal cancer screening programmes (UK: 57.4%) [23]. Like other Spanish programmes, participation was higher among women than men [6]. Healthy individuals made up 22.1% of the sample. In Spain, two surveys, nationally representative, carried out in an adult population and with data self-reported by the participants showed that 40% [24] and 16% [25] of the population did not have any of the chronic diseases on a preselected list of prevalent chronic diseases. The rate for individuals in the groups with the highest level of comorbidities (i.e., three or more dominant chronic diseases, malignancies or catastrophic conditions) was 2.9%, a figure similar to the 2.3% of bowel screening non-participants who had a medical reason for not participating in the Scottish programme [31].

To date, few studies have investigated the presence of comorbidities in people who have been invited to take part in a colorectal cancer screening programme. Furthermore, few studies have explored the effect of comorbidity on colorectal cancer screening participation and results have been controversial. Some of them highlight a positive association between screening uptake and better health status [26, 27] as it is correlated with perceived benefits that increase the likelihood of cancer screening [27]. Others show this association in the event of coexisting health problems or chronic diseases, given that these subjects contact health services more often than healthy people, and therefore can receive recommendations for screening [26, 27]. A recent systematic review focusing on facilitators and barriers to colorectal cancer screening adherence reported that individuals with a chronic disease, such as hypertension, cardiovascular disease, diabetes, arthritis, ulcers, asthma or emphysema, were more likely to participate in colorectal cancer screening, both for faecal occult blood tests and endoscopies [27]. This systematic review included seven studies specifically assessing the effect of having a chronic monitorable disease on colorectal cancer screening adherence, of which five reported the chronic disease as a significant facilitator [27]. In fact, two nationwide surveys performed in Spain based on self-reported data found significant higher compliance with colorectal cancer screening as the number of chronic conditions increased [24] or in the event of an existing comorbidity [25].

Contrary to these results, we found an inverse association between having a dominant chronic disease and completion of FIT, although we could not assess the interaction with the specific type of disease. Our results were in line with those reported by Liu et al., who found a significant negative association between three or more chronic conditions and colorectal screening (i.e., faecal occult blood test or colonoscopy) after adjusting for the number of visits to primary care [13]. Therefore, the number of medical visits should be included when the effect of comorbidity on screening participation is analysed. Some authors have hypothesized that specific differential impact of different comorbidities could explain these differences, regardless of whether there are comorbidities or not, the number of diseases, and other composite measures [12, 13, 28]. In that sense, hypertension has been associated with higher participation in colorectal cancer screening [14], whereas other studies found other chronic diseases that demand significant time with GP in the clinic for management, such as diabetes or heart disease reduced the likelihood of being up to date with screening [13]. In this regard, in our study we observed how participation is higher in individuals with multiple minor chronic diseases, possibly as they are in more frequent contact with health services compared to the healthy population and as they had a lower level of comorbidity compared to subjects who had multiple dominant chronic diseases, who participated less in the colorectal cancer screening programme.

Since recommending screening among patients with limited life expectancy for whom screening tests have little benefit and may even be harmful, older people have been a focus of research [11, 15, 29, 30]. Controversial results have been found in people aged 65 years or older with regard to the association between comorbidity and colorectal cancer screening uptake, with the results ranging from non-significant [28] to a weak association [11].

Feedback from GP suggested that some participants invited had a specific medical reason (the highest number of comorbidities) for not being screened [31]. A more holistic view will help us to determine which individuals are at an increased risk of developing colorectal cancer or its precursor lesions, how these factors influence participating in population programmes, and which individuals may benefit most from colorectal cancer screening [31]. The role of GP has been changing with the introduction of organised programmes in which they do not directly order screening tests, but rather have an essential role in increasing participation and informing the target population [32]. The primary care setting offers a chance to address colorectal cancer screening issues with patients who attend for other reasons. Taking into account background comorbidities, previous screening history, colorectal cancer risk, and individual preferences for screening could lead to a desirable informed-decision, optimizing the benefits and cost effectiveness of colorectal cancer screening. We should pay special attention to individuals with chronic dominant diseases, individualising the risk/benefit balance of screening.

### Strengths and limits

The strengths of this study include a large sample population at average risk of developing colorectal cancer, in terms of age, sex, comorbidities and socioeconomic status. The population of the 10 centres included in the RCT is representative of the population invited to the first round of screening in Barcelona based on the sociodemographic data published. Comorbidities are associated with a major use of health resources and may increase the opportunity of receiving a cancer screening recommendation from a doctor. In this study, the number of visits to primary care was also reported and appropriately analysed as a determining confounding factor. To our knowledge, this is the first study using CRG categories to assess the impact of comorbidity on a colorectal cancer screening programme. In this regard, in addition to the presence and the number of comorbidities, additional information, such as the severity of the chronic disease (i.e., minor or dominant) or its duration (i.e., acute, chronic) was given. The scientific literature is heterogeneous, since no gold standard approach exists for measuring comorbidity in the context of cancer [33]. The suitability of alternative measures may depend on the research question and also availability of data [33]. As a result, it is difficult to compare results between studies with appropriate accuracy, because the results are heavily dependent on the list of comorbidities used in each study. When reporting the effect of comorbidity on cancer screening participation, comorbidities have been measured in the literature as present or not, as a number [12, 13, 24] and/or type [13, 14] of chronic diseases, as well as using specific composite indicators (i.e., Charlson score [11]). As a limitation of the present study, we had no information about the specific type of chronic conditions or the International Classification of Diseases code, which meant we were unable to examine their interaction with the CRG categories assessed in the study. It should be pointed out that the patients excluded due to a lack of information on the comorbidity variable had a clinical profile less favourable for colorectal cancer screening participation. Nevertheless, the percentage of exclusions for this reason was small (4.6%). As expected, there was a not negligible percentage of missing lifestyle data (i.e., smoking, alcohol), especially for body mass index. This may be because weight, and hence body mass index, is widely unmeasured in primary care databases [34]. It is important to mention that this study had an urban population as the BCCSP was carried out in the city of Barcelona and more studies would be required to extrapolate these data to the entire population at average risk of developing colorectal cancer in Catalonia. This study took place in the context of a FIT population-based colorectal cancer screening programme, so the results cannot be generalised to other colorectal cancer screening tests (i.e., colonoscopy), although faecal testing is the most frequently used type of testing in Europe.

## Conclusions

Having more comorbidities, especially three or more dominant chronic diseases, was associated with statistically significant lower participation in a FIT-based colorectal cancer screening programme. On the other hand, individuals with multiple minor chronic diseases were more likely to participate in the colorectal cancer screening programme. More positive engagement by GP is required to overcome barriers and reach desirable colorectal cancer screening rates, especially among individuals who may benefit most from screening. However, at the same time, it is also important to identify patients with medical reasons for non-participation in order to reduce risks. Further research is needed to support our findings and to look into the burden of specific diseases, in addition to the presence or number of chronic diseases, on colorectal cancer screening uptake. We must continue to explore, using qualitative research, comorbidities as a reason for non-participation in colorectal cancer screening, especially in the context of organised programmes, and design specific interventions for the target population.

## Abbreviations

BCCSP: Colorectal cancer screening programme of Barcelona
CRG: Clinical risk group
FIT: Faecal immunochemical test
GP: General practitioners
